# Teaching Digital Medicine to Undergraduate Medical Students With an Interprofessional and Interdisciplinary Approach: Development and Usability Study

**DOI:** 10.2196/56787

**Published:** 2024-09-30

**Authors:** Annabelle Mielitz, Ulf Kulau, Lucas Bublitz, Anja Bittner, Hendrik Friederichs, Urs-Vito Albrecht

**Affiliations:** 1 Department of Digital Medicine Medical School OWL Bielfeld University Bielefeld Germany; 2 Department of Smart Sensors Hamburg Technical University Hamburg Germany; 3 Dean's Office for Academic Affairs Medical School OWL Bielfeld University Bielefeld Germany; 4 Department for Medical Education Medical School OWL Bielfeld University Bielefeld Germany

**Keywords:** medical education, digital medicine, digital health

## Abstract

**Background:**

An integration of digital medicine into medical education can help future physicians shape the digital transformation of medicine.

**Objective:**

We aim to describe and evaluate a newly developed course for teaching digital medicine (the Bielefeld model) for the first time.

**Methods:**

The course was held with undergraduate medical students at Medical School Ostwestfalen-Lippe at Bielefeld University, Germany, in 2023 and evaluated via pretest-posttest surveys. The subjective and objective achievement of superordinate learning objectives and the objective achievement of subordinate learning objectives of the course, course design, and course importance were evaluated using 5-point Likert scales (1=*strongly disagree*; 5=*strongly agree*); reasons for absences were assessed using a multiple-choice format, and comments were collected. The superordinate objectives comprised (1) the understanding of factors driving the implementation of digital medical products and processes, (2) the application of this knowledge to a project, and (3) the empowerment to design such solutions in the future. The subordinate objectives comprised competencies related to the first superordinate objective.

**Results:**

In total, 10 undergraduate medical students (male: n=4, 40%; female: n=6, 60%; mean age 21.7, SD 2.1 years) evaluated the course. The superordinate objectives were achieved well to very well—the medians for the objective achievement were 4 (IQR 4-5), 4 (IQR 3-5), and 4 (IQR 4-4) scale units for the first, second, and third objectives, respectively, and the medians for the subjective achievement of the first, second, and third objectives were 4 (IQR 3-4), 4.5 (IQR 3-5), and 4 (IQR 3-5) scale units, respectively. Participants mastered the subordinate objectives, on average, better after the course than before (presurvey median 2.5, IQR 2-3 scale units; postsurvey median 4, IQR 3-4 scale units). The course concept was rated as highly suitable for achieving the superordinate objectives (median 5, IQR 4-5 scale units for the first, second, and third objectives). On average, the students strongly liked the course (median 5, IQR 4-5 scale units) and gained a benefit from it (median 4.5, IQR 4-5 scale units). All students fully agreed that the teaching staff was a strength of the course. The category *positive feedback on the course or positive personal experience with the course* received the most comments.

**Conclusions:**

The course framework shows promise in attaining learning objectives within the realm of digital medicine, notwithstanding the constraint of limited interpretability arising from a small sample size and further limitations. The course concept aligns with insights derived from teaching and learning research and the domain of digital medicine, albeit with identifiable areas for enhancement. A literature review indicates a dearth of publications pertaining to analogous courses in Germany. Future investigations should entail a more exhaustive evaluation of the course. In summary, this course constitutes a valuable contribution to incorporating digital medicine into medical education.

## Introduction

### Background

Digital health, the field in which health care is linked to technology [1], encompasses digital medicine, which refers to the specific use of digital technology products in health care [1]. Following the suggestion by Bahagon and Jacobson [2] that the successful implementation of such products is “the expertise of tailoring knowledge and leadership capabilities in multidisciplinary areas: clinical, ethical, psychological, legal, comprehension of patient and medical team engagement etc...,” *digital medicine* is defined in this paper as follows: digital medicine is concerned with the holistic development and application of digital health applications, where *holistic* means that nonmedical issues and aspects relevant to such development and application are taken into account.

The World Health Organization recommends implementing digital health—and, consequently, digital medicine—technologies in the health care sector due to the potential positive impact of such technologies on health care [3]. This includes products such as health apps and smart devices for monitoring purposes and the use of technologies such as artificial intelligence, big data analysis, advanced computing, and robotics. Promising fields of application are, for example, for cardiovascular diseases [4,5], diabetes [6], and mental health [7]. Successful implementation of digital technologies is underway in many areas of health care. Still, it depends on a culture of change that results in the reorganization of services based on the public’s health needs. Among other authors, Iyamu et al [8] refer to this process as the digital transformation of medicine. As relevant stakeholders, physicians have a responsibility to shape this transformation. To prepare and enable future physicians to do so, they need to acquire relevant competencies during their studies. For the first time, skills in the field of digital medicine are classified as core competencies for the medical profession in the draft bill of the medical licensing regulations of June 2023 [9,10]. Training should enable future physicians to take an “active and self-designing role to develop a goal-oriented approach to current technological solutions on their responsibility” [11]. In Germany, the National Competency-Based Catalog of Learning Objectives (NKLM), which lists the competencies that medical students should acquire during their studies, specifies that medical students should acquire the competence of knowing and being able to reflect on the areas of application of digital medicine and the significance of digitalization. For example, they should be able to explain the application scenarios for telemedicine applications and their framework conditions [12]. Courses in digital medicine are necessary to acquire these competencies. Against this background, an integration of topics of digital health and medicine into medical education is recommended [3,11,13-15]. It has already been shown that medical students feel like the early integration of such topics into medical education can help prepare them for their future work environment [16].

Courses on digital health are already offered as part of medical studies in various countries, including Germany [17,18], but the range of classes dealing with digital medicine must be further expanded to enable a true digital transformation of the health care system. Studies such as those by Jacob et al [11], Schreiber et al [15], and Machleid et al [16] show that digital medicine has so far not been given sufficient consideration in medical education. Thus, it can be assumed that future physicians do not yet have the required digital medicine competencies. Many students feel unprepared for the future regarding the digitalization of health care by their teachers and the courses they attended and wish for better integration of digital medicine and digital health topics into their studies [11,16,19]. Against the background of the integration of competencies in digital medicine in the draft bill of the medical licensing regulations of June 2023 [10], an expansion of courses related to digital medicine in Germany may be expected as soon as the new medical licensing regulations come into force.

Following the call for corresponding adjustments, the newly founded Medical School Ostwestfalen-Lippe (OWL; the region of Ostwestfalen-Lippe, Germany) at Bielefeld University has set up a Digital Medicine working group within the faculty and included Digital Medicine in the regular curriculum of medical studies. In addition, a Technological Transformation in Medicine profile is offered for continuing education. In this profile, the Digital Medicine working group contributes with a course called Digital Medicine, referred to in this paper as the “Bielefeld model.” The overarching educational objective is to empower students to feel able to actively engage as prospective architects of digital medicine, transcending their role as mere users or consumers of digital products or participants in digital processes. This means that students will need to be equipped to develop and use digital applications comprehensively.

This overarching educational objective translates to 3 specific superordinate learning objectives of the course (Textbox 1).

**The 3 superordinate learning objectives of the course.**

**Superordinate learning objective 1**

Students will know the factors influencing the sustainable implementation of digital medical products and processes.

**Superordinate learning objective 2**

Students will be able to apply their knowledge of the factors influencing the sustainable implementation of digital medical products and processes in developing a specific project.

**Superordinate learning objective 3**

Students will feel empowered to design sustainable digital products and processes in future projects.

The implementation of digital products and processes is considered to be sustainable if it is independent of individual motivated implementers. To design sustainable solutions, it is important to consider a wide range of influencing factors. Only if these are taken into account will products and processes be able to achieve a high level of acceptance in the health care system in the long term, develop their full positive impact, and remain in place.

The first of these 3 superordinate learning objectives, in turn, translates to 17 subordinate learning objectives (Textbox 2). These subordinate learning objectives include abstract competencies related to the first superordinate learning objective (subordinate learning objectives 1 to 6) as well as specific competencies in relation to the individual factors that are subsumed under the first superordinate learning objective (subordinate learning objectives 7 to 17). Thus, these subordinate learning objectives should capture aspects of the first superordinate learning objective.

**The 17 subordinate learning objectives of the course.**

**Subordinate learning objective ID and description**

- Sub01: the students can identify players and stakeholders in the field of digital medicine.
- Sub02: the students can name and reflect on overarching themes of digital medicine.
- Sub03: the students can identify, address, and discuss problem areas (medical, technical, legal, ethical, and social) of digital medicine.
- Sub04: the students can critically assess digital medicine and evaluate it based on its opportunities and challenges.
- Sub05: the students can explain the product life cycle of digital medicine and plan an applied project based on it.
- Sub06: the students can plan a concrete project with basic knowledge of project management.
- Sub07: the students can identify markets for digital medicine and discuss challenges of market entry for digital applications.
- Sub08: the students can identify and communicate the necessary information required for commissioning a technical development of digital applications.
- Sub09: the students can explain the legal challenges of using digital tools and digital communication media in relation to digital medicine.
- Sub10: the students can explain the regulatory difference between a medical device and other products and explain legal consequences.
- Sub11: the students can identify and discuss the ethical implications of digital medicine for patients, physicians, contributors, society, and the environment.
- Sub12: the students can explain interoperability and know what characterizes interoperability.
- Sub13: the students can assess the quality of digital medicine applications.
- Sub14: the students can recognize the quality of the usability of a digital application and know which factors can influence the usability.
- Sub15: the students can discuss elements of data science and its tools and requirements in data preparation and data analysis.
- Sub16: the students can discuss and evaluate digital medicine with regard to specific aspects in the context of gender and sex.
- Sub17: the students can explain data protection and data security challenges in the development and use of digital tools and digital communication media.

The decision to design an own course concept instead of adopting an existing one was made to be able to design a course that is very specifically geared toward achieving these learning objectives.

### Objectives

The aim of this paper is to introduce the Bielefeld model for the first time and undertake an initial exploratory evaluation.

In particular, the aim of the course evaluation is to determine the following:

1. How well the participants objectively and subjectively achieved the 3 superordinate learning objectives (as presented in Textbox 1) and how well they objectively achieved the 17 subordinate learning objectives of the course (as presented in Textbox 2).
2. Whether the course was well designed. This encompasses how suitable the participants considered the course to be for achieving the superordinate learning objectives, whether they enjoyed the course and benefitted from it, and which aspects they considered as strengths of the course.
3. How important the course was to the participants. This encompasses how important the achievement of the superordinate learning objectives was to them personally and the reasons for the participants’ potential absences during the course. Those reasons might indicate whether taking part in the course was important for the participants.

## Methods

### The Digital Medicine Course

#### Setting

Undergraduate medical students at the Medical School OWL go through a model curriculum with an interdisciplinary specialization in their first 3 years of studies. The students can choose, or, in rare cases, are assigned to 1 of 5 profile options with Technological Transformation in Medicine (TeTraMed) being one of them. Digital Medicine is the third of the 6 mandatory modules the TeTraMed profile comprises. The module is completed with an examination. Students can earn a bachelor’s degree in Interdisciplinary Medical Sciences alongside their medical studies, with course credits counting toward both degrees.

#### Course Concept

The instructors of the course designed the course and developed the learning objectives based on their experience in research and teaching in digital medicine and in the development of digital medicine applications and based on considerations for the design of an effective course concept. The main instructor is a physician, computer scientist, and public health scientist and a professor of digital medicine at Bielefeld University. Coinstructors (a psychologist, physician, and medical engineer) were members of this working group. The overarching goals and learning objectives of the course were to be achieved by the students by developing a digital medicine application in interdisciplinary collaboration with electrical engineering students from Hamburg University of Technology (TUHH; when students are mentioned hereafter in connection with the course, this refers to medical students. Electrical engineering students are only referred to when they are specifically mentioned. When mentioning the course, unless otherwise stated, this refers to the course for medical students). The course was co-designed with a professor of smart sensors who is skilled in computer science and electrical engineering and teaches the electrical engineering students at TUHH. Consistent with the 3 superordinate learning objectives (Textbox 1), the didactic emphasis of the course was directed toward investigating the manifold factors associated with and influencing the domain of digital medicine. Adhering to project-based learning methodologies, medical students collaboratively engaged in small groups in their own projects related to telemedicine (using technologies for providing health care over a distance [20]), an area of digital medicine. The students were divided into 3 groups, and each group was assigned 1 of 3 cases. The cases described circumstances in which individuals required monitoring due to medical conditions or circumstances (for more detail, see Multimedia Appendix 1). The aim of the students was to systematically solve the challenge described in the case by using mobile sensor technology, exemplifying its application in a corresponding product and process. They were also free to design an accompanying app. A course that was separate but linked to the course for medical students was held at TUHH with the electrical engineering students. Their course followed a research-based learning design. The focus of the electrical engineering students was primarily on developing the sensor systems that the medical students designed in their projects. The sensor should have been designed by the end of the course, but the aim was not to actually implement it on the market. The collaboration between the courses at TUHH and Bielefeld University was designed to emulate the fact that, when working on projects such as that in the Bielefeld model in real life outside the university, a collaboration between medical professionals and technicians is often required to technically implement what medical professionals design. The central concept involved both medical and electrical engineering students collaborating without silos, working together as equals. This approach fosters interdisciplinary cooperation, promotes precise and accurate communication, and equips students for project work in professional settings across both fields. Apart from this collaboration, the medical students and the electrical engineering students were taught separately. The electrical engineering students did not take part in the regular sessions of the Bielefeld model.

The students experienced the entire product life cycle within the Digital Medicine course, from brainstorming to planning, developing, evaluating, implementing, operating, and decommissioning digital products and processes. They were confronted with challenges posed by the diverse interests of the stakeholders, frameworks, resources, and settings involved and had to take into account the factors influencing the sustainable implementation of digital medical products and processes. This encompassed an exploration of their interrelationships and their consequential impact on the development and enduring implementation of digital medical products and processes. By doing so, they practiced directly acknowledging, accepting, and addressing these factors.

On the basis of their experience, the instructors identified and addressed the following multidimensional factors in the course: project management, market entry, technical development, quality, data science, interoperability, usability, law, regulation and ethics, sex and gender sensitivity, and data protection and security. These factors result from the interfaces between medicine and health with the fields of technology, informatics, society, law, regulation, ethics, economics, and psychology.

In each session, one or several of these factors were addressed by the course instructors and often also by external experts who were invited as guest lecturers (Table 1; see Multimedia Appendix 2 for more detailed information about the topics of the sessions and the guest lecturers). Table 1 shows which of the 17 subordinate learning objectives relate to the specific sessions. It was assumed that subordinate learning objectives 1 to 6 (Textbox 2) would be achieved throughout the course. The subordinate learning objectives that relate specifically to the factors subsumed under the first superordinate learning objective (subordinate learning objectives 7 to 17; Textbox 2) should be achieved in the corresponding sessions. After the lecture in which the factor or factors were addressed, the students connected the topic to their own projects in a practical unit, reflecting on its impact and identifying what considerations were necessary for their specific work. For example, in the session on law, regulation, and ethics, students were able to consider whether their sensor would be a medical device from a legal point of view, or in the session on usability, for example, they were able to reflect on how usable their product was and how usability could be increased. The students could exchange ideas, discuss, develop further content, and converse about general and project-related questions with the lecturers alone or in a group. In this process, the students identified and named requirements that their sensors should fulfill. They communicated the technical requirements to the electrical engineering students in Hamburg, who implemented these specifications for the sensors in consultation with the medical students. Progress and outcomes of the small groups were systematically documented in a project outline (as illustrated in Multimedia Appendix 3) subject to continuous updates and revisions throughout the course.

**Table 1:** Digital Medicine curriculum within the TeTraMed profile.

Session	Topic	Life cycle phase	Subordinate learning objective to be achieved in the session	Guest lecturer
1	Introduction	Idea generation	—^a^	No
2	Project management	Planning	6	Yes
3	Market entry	Planning	7	Yes
4	Technical development	Development	8	Yes
5	Quality^b^	Development	13	No
6	Data science^c^	Development	15	Yes
Additional session A	Meeting with the electrical engineering students	Development	8	—
7	Interoperability	Development	12	Yes
8	Usability	Premarket evaluation	14	Yes
9	Law, regulation, and ethics	Implementation	9, 10, and 11	Yes
10	Sex and gender sensitivity	Postmarket evaluation	16	Yes
11	Data protection and data security	Use	17	No
12	End	Decommissioning	—	No
Additional session B^d^	Ceremony	Memorial	—	—

^a^Not applicable.

^b^Deviation from the original curriculum (Law, Regulation, Ethics) was changed following the students’ wishes as it conflicted with other study responsibilities.

^c^Deviation from the original curriculum (Interoperability) was changed following the students’ wishes as it collided with other study liabilities.

^d^In this session, it was planned to hand the participants certificates that confirmed participation in the course. This session had to be canceled due to situational circumstances, but the certificates were handed out anyway.

By confronting new issues and challenges, students were encouraged to reflect on their own project on an ongoing basis and then revise it. The course instructors and external experts from the different interface areas provided knowledge transfer and support for the projects. The course concept provided self-management skills (managing oneself and the available resources) as well as project and team management skills, such as preparing, implementing, and recording project meetings. Work, communication, interaction, negotiation, and conflict resolution in interdisciplinary, international, and intercultural teams and exchange and work in English were also part of this. The course was primarily offered in an analog-oriented format; essentially, only the meetings with the electrical engineering students took place in web-based live video formats. The course was supported by web-based materials provided on the university e-learning platform LernraumPlus. Due to external organizational circumstances, some sessions were only available on the web for self-study.

#### Course Structure

The Digital Medicine course was structured in 12 sessions, usually held weekly, distributed over 4 months in the summer semester of 2023 (April to July). Each session consisted of a 45-minute lecture, a 90-minute workshop during which the students worked on their projects, and a 90-minute guided self-study for which no contact time with the teaching staff was scheduled but was offered at times. Thus, there were a total of 9 hours of lectures, 18 hours of workshops, and 18 hours of guided independent study. In addition, times without contact with the teachers were set aside for preparation and follow-up work. In total, 3 further scheduled dates were canceled due to holidays. The structure of each course day is shown in Multimedia Appendix 4.

### Evaluation and Analysis

#### Overview

The course concept was evaluated by recruiting undergraduate medical students at the Medical School OWL who took part in the course between April and July 2023. The students were asked to complete a web-based presurvey at the beginning of the course and a postsurvey after the course had ended (after the 12th session) in German using a customized web-based survey tool. For this, they first had to create a pseudonym. The mapping between the pseudonyms and the participants was unknown to the study staff, so anonymity was maintained. However, using the pseudonyms made it possible to merge the individual data sets of the pre- and postsurveys. A customized instrument (using the formr survey framework [21]) was provided for the survey by the Medical Education working group at the Medical School OWL at Bielefeld University. The presurvey was administered on April 3, 2023, and the postsurvey was administered between July 10, 2023, and August 1, 2023. Students were invited anonymously via email and by the instructors during the course. A total of 2 follow-up actions were conducted via email at an interval of 2 weeks after the beginning of the postevaluation.

#### Instruments

#### Overview

The presurvey consisted of 27 items: the demographic variables of age and gender, the items related to the objective achievements of the subordinate learning achievements (pre- and postsurvey), as well as the open comments (again, pre- and post-survey, see corresponding tables in the Results section), and an additional 10 items that will not be described or analyzed in this paper but will be focused on in a separate publication. These additional items were administered to assess the achievement of learning goals following a catalog of learning outcomes as described by Foadi et al [22] that partially corresponds to the NKLM. The postsurvey consisted of 47 items and additional 10 items based on the learning outcomes as described by Foadi et al [22] that are neither described nor evaluated in this paper. A comprehensive overview of all items that were used can be found in Multimedia Appendix 5.

All responses to the surveys (except regarding items AB01 and COM01, as described later in the manuscript when these items are presented) were recorded on an ordinal 5-point Likert scale, which was recoded after the study to be more consistent with the usual coding system (the coding in the questionnaire was as follows: 1=*strongly agree*, 2=*rather agree*, 3=*rather neutral*, 4=*rather disagree*, and 5=*strongly disagree*; the scale was recoded as follows for the analyses: 1=*strongly disagree*, 2=*rather disagree*, 3=*rather neutral*, 4=*rather agree*, and 5=*strongly agree*). All items (except those by Foadi et al [22]) were developed in-house, as described in detail in the following sections.

#### Achievement of the Super- and Subordinate Learning Objectives

The objective and subjective achievement of the 3 superordinate learning objectives was assessed using 1 item each in the postsurvey (for objective achievement: items SUPER1_OA, SUPER2_OA, and SUPER3_OA; for subjective achievement: items SUPER1_SA, SUPER2_SA, and SUPER3_SA). The objective achievement of the subordinate learning objectives was assessed using 17 items (items sub01 to sub17). By recording the objective achievement of the subordinate learning objectives in both the pre- and the postsurvey, it was possible to determine whether the participants would have achieved these objectives better after the course than before.

As the subordinate learning objectives should capture aspects of the first superordinate learning objective, items measuring the objective achievement of these subordinate objectives should capture aspects of the objective achievement of this first superordinate learning objective. In that case, changes in the median of items measuring the objective achievement of the subordinate learning objectives from the pre- to the postsurvey would also indirectly provide information about how the objective achievement of the first superordinate learning objective developed from the pre- to the postsurvey.

#### Design of the Course

One item per superordinate learning objective was used to record the extent to which the participants considered the course concept suitable for achieving this objective (items SUPER1_SUIT, SUPER2_SUIT, and SUPER3_SUIT). In total, 2 items were used to measure whether the participants enjoyed the course (item FUN01) and whether they felt that they benefited from it (item BEN01). In 4 questions, participants were able to indicate the extent to which they perceived 4 potential benefits as actual strengths of the course (items STR01 to STR04).

#### Importance of the Course to the Participants

One item per superordinate learning objective was used to record how important it was to the participants to achieve this objective (items SUPER1_IMP, SUPER2_IMP, and SUPER3_IMP).

One item was used to measure the reasons for participants’ absences (item AB01) during the course. This item did not use a Likert scale but instead offered various answer options for participants to choose from, allowing them to give multiple answers.

#### Open Comments

Participants could write comments in an open-response format at the end of the pre- and postsurvey (item COM01).

#### Development, Validity, and Reliability of the Instruments

All items were developed through an iterative process informed by the insights of 3 authors. The items assessing the objective achievement of the superordinate (items SUPER1_OA, SUPER2_OA, and SUPER3_OA) and subordinate (all items) learning objectives were developed by formulating the learning objectives in first-person singular format and pairing them with a 5-point Likert scale. The development of the learning objectives and the items based on them was carried out by 2 (in the case of the learning objectives) to 3 (in the case of the items) authors in an iterative process. The consistency of the formulations with the course’s conceptual objectives was regularly reviewed to ensure strong content validity. The reason for the development of these new items was that, until now, no instrument has reflected the course concept closely enough to capture what was to be learned in the course. The content validity of the other items was also ensured by matching the formulations in an iterative process with the underlying idea of what should be captured using these items. The items assessing whether the participants enjoyed the course (item FUN01) and whether they benefitted from it (item BEN01) have been used with similar or related wordings in other studies [23,24]. Therefore, content validity was assumed.

The items did not capture common things but were instead considered individually, so they were not combined as a scale. Thus, no internal consistencies were calculated. Interrater reliability was assumed for all items that were recorded and statistically evaluated on the Likert scale as the evaluation was objectively independent of the rater.

#### Analysis

All items were analyzed descriptively. The mean and SD were calculated to describe the age-related data. The information on gender was analyzed through the calculation of occurrence frequencies. The Spearman ρ was calculated to investigate a correlation between the item measuring the objective achievement of the first superordinate learning objective and the 17 items measuring the objective achievement of the subordinate learning objectives in the postsurvey. A positive correlation would indicate that the items measuring the objective achievement of the subordinate learning objectives are well suited to capture aspects of the objective achievement of the first superordinate learning objective. The items that were recorded using the ordinal 5-point Likert scales were evaluated by calculating the median and IQR. Using a descriptive account of the aggregated median values of the students for the items assessing the objective achievement of the subordinate learning objectives, we compared a possible change in this achievement from the pre-to the postsurvey. Textbox 3 shows how the changes in the median of these items from the pre- to the postsurvey will be evaluated qualitatively. The qualitative evaluation was based on considerations of how changes are to be evaluated. Due to the small sample, an inferential statistical analysis was not performed. The comments were analyzed by dividing them into main categories and subcategories based on the structuring qualitative content analysis according to Kuckarzt and Rädiker [25]. The frequency of occurrence of each subcategory was determined.

**Qualitative evaluation of the changes in the median of the objective achievement of the subordinate learning objectives.**

**Size of change and qualitative evaluation**

- 0: no change
- >0 to 0.4: minimal change
- 0.5 to 0.9: small change
- 1 to 1.4: rather big change
- 1.5 to 1.9: big change
- >1.9: extensive change

### Ethical Considerations

The study was approved by the Ethics Committee Ethik-Kommission Westfalen-Lippe, located in Münster, Germany, under the chairmanship of Professor Dr Wolfgang E Berdel, on May 11, 2023 (2023-233-f-S).

## Results

All 15 course participants were recruited for the study. A total of 10 participants (n=4, 40% male and n=6, 60% female; mean age 21.7, SD 2.1 years) completed both the pre- and the postsurvey and were included in the analysis.

### Achievement of the Super- and Subordinate Learning Objectives

Table 2 shows the results regarding the objective and subjective achievement of the superordinate learning objectives (for information on how frequently the individual response categories were selected by the participants, see Multimedia Appendix 6). Regarding the objective achievement of these objectives, medians of 4 (IQR 4-5), 4 (IQR 3-5), and 4 (IQR 4-4) scale units were found for the first, second, and third superordinate learning objectives, respectively. Concerning the question of whether the participants also subjectively achieved these learning objectives, medians of 4 (IQR 3-4), 4.5 (IQR 3-5), and 4 (IQR 3-5) scale units were found for the first, second, and third learning objectives, respectively. Therefore, the median values varied between the scale points *rather agree* (scale value of 4) and *strongly agree* (scale value of 5). Thus, on average, the results indicate a rather to very good achievement of the 3 superordinate learning objectives both objectively and subjectively.

**Table 2:** Objective and subjective achievement of the 3 superordinate learning objectives, the suitability of the course for achieving them, and the importance of achieving them, administered in the postsurvey (N=10)^a^.

Superordinate learning objective and item ID		Item	Scale units, median (IQR)
**Superordinate learning objective 1**			
	SUPER1_OA^b^	I know the factors that influence the sustainable implementation of digital medical products and processes.	4 (4-5)
	SUPER1_SUIT^c^	The course concept was suitable for teaching these factors.	5 (4-5)
	SUPER1_IMP^d^	It was important to me to achieve this learning goal.	4 (3-4)
	SUPER1_SA^e^	I have achieved this learning goal from a personal perspective.	4 (3-4)
**Superordinate learning objective 2**			
	SUPER2_OA^f^	I can apply my knowledge regarding the factors that influence the sustainable implementation of digital medical products and processes in developing a concrete project.	4 (3-5)
	SUPER2_SUIT	The course concept was suitable to apply my knowledge regarding these factors in developing a concrete project.	5 (4-5)
	SUPER2_IMP	It was important to me to achieve this learning goal.	4 (3-5)
	SUPER2_SA	I have achieved this learning goal from a personal perspective.	4.5 (3-5)
**Superordinate learning objective 3**			
	SUPER3_OA^f^	I feel empowered to design sustainable digital products and processes in future projects.	4 (4-4)
	SUPER3_SUIT	The course concept was suitable to enable me to design sustainable digital products and processes in future projects.	5 (4-5)
	SUPER3_IMP	It was important to me to achieve this learning goal.	3.5 (3-5)
	SUPER3_SA	I have achieved this learning goal from a personal perspective.	4 (3-5)

^a^The “superordinate learning objective” column indicates to which superordinate learning objective the respective items belong.

^b^OA: objective achievement (of the respective superordinate learning objective).

^c^SUIT: suitability (of the course concept for achieving the respective superordinate learning objective).

^d^IMP: importance (of achieving the respective superordinate learning objective).

^e^SA: subjective achievement (of the respective superordinate learning objective).

^f^This item was answered by only 90% (9/10) of the participants.

Regarding the objective achievement of the subordinate learning objectives, on average, the participants performed better on all items after the course than before (Table 3; for information on how frequently the individual response categories were selected by the participants, see Multimedia Appendix 7). While an average median of 2.5 (IQR 2-3) scale units was achieved across the items in the presurvey, it increased by 1.5 scale units to 4 (IQR 3-4) in the postsurvey. This can be rated as a big change (Textbox 3). While, in the presurvey, the median values varied between the scale points *strongly disagree* (scale value of 1) and *rather agree* (scale value of 4), in the postsurvey, they varied between the scale points *rather neutral* (scale value of 3) and *strongly agree* (scale value of 5). While the presurvey results, therefore, indicate a strong nonachievement to rather good achievement on average of the subordinate learning objectives, the postsurvey results indicate a partial to very good achievement of those objectives on average. When analyzing the change in the median for each session, there was an improvement from the pre- to the postsurvey in 15 of the 17 items (Table 3; range of change in the median between 0.5 and 2 scale units). For items sub03 and sub04, no change in the median values could be found. Still, when looking at how often the individual response categories were selected (Multimedia Appendix 7), it can be seen that, in these items, there was also an improvement from the pre- to the postsurvey. While only 60% (6/10) of the participants strongly or rather agreed with items sub03 and sub04 in the presurvey, 100% (10/10) of the participants strongly or rather agreed with these items in the postsurvey. It can be summarized that, on average, all subordinate learning objectives were achieved better after the course than before by the participants.

**Table 3:** Objective achievement of the subordinate learning objectives, administered in the pre- and postsurvey (N=10).

Item ID	Item	ρ^a^	Presurvey score, median (IQR)^b^	Postsurvey score, median (IQR)^c^	∆Median^d^
Sub01	I can identify players and stakeholders in the field of digital medicine.	0.37	2 (1-3)	4 (3-5)	2
Sub02	I can name and reflect on overarching themes of digital medicine.	0.56	3 (2-4)	4.5 (4-5)	1.5
Sub03	I can identify, address, and discuss problem areas (medical, technical, legal, ethical, and social) of digital medicine.	0.11	4 (3-5)	4 (4-5)	0
Sub04	I can critically assess digital medicine and evaluate it based on its opportunities and challenges.	0.30	4 (3-4)	4 (4-5)	0
Sub05	I can explain the product life cycle of digital medicine and plan an applied project based on it.	0.73	2 (1-2)	4 (3-4)	2
Sub06	I can plan a concrete project with basic knowledge of project management.	0.85	2 (1-2)	4 (3-5)	2
Sub07	I can identify markets for digital medicine and discuss challenges of market entry for digital applications.	0.14	3 (1-3)	4 (3-5)	1
Sub08	I can identify and communicate necessary information required for commissioning a technical development of digital applications.	0.42	2 (2-3)	4 (4-4)	2
Sub09	I can explain the legal challenges of using digital tools and digital communication media in relation to digital medicine.	0.56	3 (2-3)	4 (4-4)	1
Sub10	I can explain the regulatory difference between a medical device and other products and explain legal consequences.	0.52	3 (2-3)	4 (4-5)	1
Sub11	I can identify and discuss the ethical implications of digital medicine for patients, physicians, contributors, society, and the environment.	0.65	3 (3-4)	3.5 (3-4)	0.5
Sub12	I can explain interoperability and know what characterizes interoperability.	0.79	1.5 (1-3)	3.5 (3-4)	2
Sub13	I can assess the quality of digital medicine applications.	0.60	3 (2-3)	4 (4-5)	1
Sub14	I can recognize the quality of the usability of a digital application and know which factors can influence the usability.	0.38	2 (1-3)	4 (4-5)	2
Sub15	I can discuss elements of data science and its tools and requirements in data preparation and data analysis.	0.62	2 (1-3)	3 (2-4)	1
Sub16	I can discuss and evaluate digital medicine with regard to specific aspects in the context of gender and sex.	0.50	2 (1-3)	4 (3-4)	2
Sub17	I can explain data protection and data security challenges in the development and use of digital tools and digital communication media.	0.44	2.5 (2-3)	4 (3-4)	1.5

^a^Spearman ρ (correlation between the item measuring the objective achievement of the first superordinate learning objective and the items measuring the objective achievement of each subordinate learning objective as measured in the postsurvey).

^b^Average median 2.5 (IQR 2-3).

^c^Average median 4 (IQR 3-4).

^d^Average change in the median from the pre- to the postsurvey: 1.5 (IQR 1-2).

We found a mostly medium to high correlation between the item measuring the objective achievement of the first superordinate learning objective and the 17 items measuring the objective achievement of the subordinate learning objectives (Table 3). This suggests that these items are well suited to capture aspects of the objective achievement of the first superordinate learning objective. Therefore, these results indirectly suggest that achieving the first superordinate learning objective might have also improved from the pre- to the postsurvey.

Regarding the size of the changes in the median, rather big, big, or extensive changes were found for 14 of the 17 items (Tables 3 and 4). The smallest (small) change was observed in item sub11 (Tables 3 and 4). The greatest (extensive) changes were observed in items sub01, sub05, sub06, sub08, sub12, sub14, and sub16 (Tables 3 and 4).

**Table 4:** Evaluation of the size of the change in the median from the pre- to the postsurvey regarding the 17 items measuring the objective achievement of the subordinate learning objectives (N=17).

Qualitative evaluation	Items, n (%)	Item ID
No change	2 (12)	Sub03 and sub04
Minimal change	0 (0)	—^a^
Small change	1 (6)	Sub11
Rather big change	5 (29)	Sub07, sub09, sub10, sub13, and sub15
Big change	2 (12)	Sub02 and sub17
Extensive change	7 (41)	Sub01, sub05, sub06, sub08, sub12, sub14, and sub16

^a^Not applicable.

In addition, as seen in Table 5, there was an average improvement of the median across all 17 items for each participant from the pre- to the postsurvey (range of change in the median between 1 and 3 scale units; in addition, Multimedia Appendix 8 shows the raw data for each participant for each item in the pre- and postsurvey and how those values changed from the pre- to the postsurvey). To summarize, this means that all participants improved from the pre- to the postsurvey across all these learning objectives.

**Table 5:** Intraindividual differences in the median from the pre- to the postsurvey in the objective achievement of the 17 subordinate learning objectives (items sub01 to sub17; N=10).

Participant number	Presurvey score, median (IQR)	Postsurvey score, median (IQR)	∆Median^a^
1	3 (2-3)	4 (4-4)	1
2	3 (2-4)	5 (4-5)	2
3	1 (1-1)	3 (2-4)	2
4	2 (1-4)	4 (4-5)	2
5	3 (2-4)	4 (4-5)	1
6	3 (3-4)	5 (4-5)	2
7	1 (1-1)	4 (3-4)	3
8	3 (2-4)	4 (3-4)	1
9	3 (2-3)	4 (3-4)	1
10	2 (2-3)	4 (3-4)	2

^a^Change in the median from the pre- to the postsurvey.

### Design of the Course

#### Suitability of the Course for Achieving the Superordinate Learning Objectives

Concerning the question of whether the course concept was suitable for achieving the 3 superordinate learning objectives, we found a median of 5 (IQR 4-5) scale units for the first, second, and third superordinate learning objectives (Table 2; for information on how frequently the individual response categories were selected by the participants, see Multimedia Appendix 6). The median values correspond to the scale point *strongly agree* (scale value of 5). Thus, the results indicate that, based on the average ratings, the course concept was strongly suited for achieving the 3 superordinate learning objectives.

#### Enjoyment of the Course

Concerning the question of whether the participants enjoyed the course, the median was 5 (IQR 4-5) scale units; for information on how frequently the individual response categories were selected by the participants, see Multimedia Appendix 6. This corresponds to the scale point *strongly agree* (scale value of 5). Therefore, this result indicates that, on average, participants enjoyed the course very much.

#### Benefits Obtained From the Course

Concerning the question of whether the participants felt that they obtained a benefit from having taken part in the course, the median was 4.5 (IQR 4-5) scale units; for information on how frequently the individual response categories were selected by the participants, see Multimedia Appendix 6. This median varies between the scale points *rather agree* (scale value of 4) and *strongly agree* (scale value of 5). Thus, this result indicates that, on average, participants obtained a benefit rated as rather to very well from taking part in the course.

#### Strengths of the Course

With regard to the question of which of several proposed aspects were strengths of the course, the median for all proposed aspects fluctuated between the scale points *rather agree* (scale value of 4) and *strongly agree* (scale value of 5; Table 6); medians of 5 (IQR 5-5), 5 (IQR 4-5), 5 (IQR 3-5), and 4 (IQR 4-5) scale units for items STR01, STR02, STR03, and STR04, respectively; for information on how frequently the individual response categories were selected by the participants, see Multimedia Appendix 6. Regarding the teaching staff, all the students strongly agreed that this was a strength, as shown in Multimedia Appendix 6. Thus, in total, the results indicate that, on average, all the characteristics mentioned were considered to be strengths of the course either somewhat or strongly.

**Table 6:** Strengths of the Digital Medicine course, administered in the postsurvey (N=10).

Item stem and ID			Item		Scale units, median (IQR)
**Strengths of the** **Digital Medicine** **course**					
	STR01^a^	The teaching staff (friendliness, openness, appreciation, professionalism, and interdisciplinarity)		5 (5-5)	
	STR02	The design of the course (content preparation, interaction, material, and equipment)		5 (4-5)	
	STR03	Timing of the classes (punctuality and time frame for lectures and seminars).		5 (3-5)	
	STR04	Offline content and preparation in the LernraumPlus platform		4 (4-5)	

^a^STR: strengths (of the course).

### Importance of the Course to the Participants

#### Importance of Achieving the Superordinate Learning Objectives

Regarding the question of whether it was important to the participants to achieve the 3 superordinate learning objectives, medians of 4 (IQR 3-4), 4 (IQR 3-5), and 3.5 (IQR 3-5) scale units were found for the first, second, and third learning objectives, respectively (Table 2; for information on how frequently the individual response categories were selected by the participants, see Multimedia Appendix 6). These median values vary between the scale points *rather neutral* (scale value of 3) and *rather agree* (scale value of 4). Therefore, the results indicate that the participants varied, on average, between being neutral about the importance of the course to them and rather agreeing that it was important to them to achieve the 3 superordinate learning objectives.

#### Reasons for Potential Absences During the Course

The reasons for not attending individual course sessions (Table 7) comprised insufficient time capacity due to other study-related requirements (10/10, 100% of the participants), illness (5/10, 50% of the participants), inadequate time capacity due to personal requirements (2/10, 20% of the participants), and perceived irrelevance for the individual participant (1/10, 10% of the participants). No participants named another reason.

**Table 7:** Reasons for nonparticipation, administered in the postsurvey (N=10).

Item ID, question, and item		Participants, n (%)
**AB01^a^: If I was unable to attend course days, it was largely due to the following reasons (multiple choices possible):**		
	Too little time capacity due to other study-related requirements	10 (100)
	Insufficient time capacity due to personal demands	2 (20)
	Illness	5 (50)
	The course was irrelevant to me	1 (10)
	Other	0 (0)

^a^AB: absence (during course sessions).

### Open Comments

There was 1 open comment in the presurvey and 5 open comments in the postsurvey. Table 8 shows the categories that appeared in the comments and the frequency of the occurrence of each subcategory. Most of the comments belonged to the main category (*positive feedback on the course or positive personal experience with the course*).

**Table 8:** Open comments (COM), administered in the pre- and postsurvey (N=5).

Item ID, item, survey, main category, and subcategory						Participants, n (%)
**COM01:** **Here you can enter further comments, suggestions, or proposals**						
	**Presurvey**					
		Request for similar workload to that of other courses of the profile			1 (20)	
	**Postsurvey**					
		**Positive feedback on the course or positive personal experience with the course**				
			The course was fun	1 (20)		
			The teaching was good	1 (20)		
			The teachers were friendly	1 (20)		
			The course contributed positively to personal and professional development or getting something out of the course	2 (40)		
			Good well-being	1 (20)		
			Gratitude for the course or for the opportunity to participate in the course	1 (20)		
		**Negative feedback on the course or negative personal experience of the course**				
			The course was only offered in person but not in a hybrid format	1 (20)		
			Stress, frustration, and a guilty conscience toward the TUHH^a^ due to the fact that one could not invest more time in the course because of limited capacity	1 (20)		
			Demotivation due to the absence of other members of their own small group	1 (20)		
		**Other**				
			Wish that one could have participated more, but this was not possible	2 (40)		
			Frequent nonparticipation due to illness	1 (20)		

^a^TUHH: for Hamburg University of Technology.

## Discussion

### Achievement of the Learning Objectives and Design of the Course

This paper presents a newly developed course concept for teaching digital medicine in medical education (the Bielefeld model) and an initial evaluation of this concept. This evaluation was based on feedback provided by undergraduate medical students who took part in this course. By developing and implementing this course, we responded to the calls [3,11,13-15] for integrating the topic of digital health or digital medicine into medical education. According to the students’ self-report in the evaluation, they were overall able to achieve the super- and subordinate learning objectives of the course and felt that the course was largely suitable for achieving them.

The evaluation results indicate that, on average, the participants achieved the super- and subordinate learning objectives of the course rather to very well, as recorded after the course. The average change in the objective achievement of the subordinate learning objectives from before to after the course could be rated as big.

The fact that there was only a small change in item sub11 could be related to the circumstance that the participants were supposed to achieve the learning objective captured in this item by autonomously working on it using web-based materials, which they may not have done.

The findings on the achievement of the learning objectives indicate that the course concept was well suited for achieving them and could be interpreted as the course being well designed. This aligns with the finding that, on average, participants perceived the course concept as well suited for attaining the 3 superordinate learning objectives. On the basis of these outcomes, it can be assumed that the course effectively fulfills its overarching objective: empowering participants to feel able to actively engage as future architects of digital medicine, transcending the role of mere participants in digital processes or users of digital products to being able to develop and use such products comprehensively.

The assumption that the course was well designed is also supported by the fact that most to all participants enjoyed the course, benefited from it, and agreed that the course’s design and teaching staff were strengths of the course. As outlined, the teaching staff consisted of interdisciplinary experts on the topics covered in the course in addition to the instructors from the Digital Medicine working group. Whether it was this interdisciplinarity, the appearance of the teaching staff (eg, friendliness, openness, and appreciation), or both that the students experienced as a strength is not clear from the results. These results are supported by positive statements in the open comments that relate precisely to the aforementioned points. The statements that the participants felt comfortable in the course and were appreciative of their participation support the overall positive evaluation of the course. However, there was also criticism that the course was not offered in a hybrid form. In the future, this may be resolved by offering hybrid courses if necessary. Reasons for the statements that participation in the course resulted in stress, frustration, or demotivation were a lack of time and the unplanned absence of course members, over which the teachers had no influence. However, should this negative experience also occur in future courses, the lecturers and students should jointly consider measures to remedy the situation. A further quality assurance evaluation by the Medical School OWL supports the overall positive assessment of the course concept. A total of 50% (5/10) of the students took part in this evaluation, and they agreed that the instructors fostered a positive learning environment, acknowledged the participants’ previous knowledge, and explained concepts clearly. These students offered comments that, in terms of content, closely aligned with those from the evaluation that is the subject of this paper, addressing both the positive and negative aspects. Unsystematic, spontaneous observations of the instructors during the course support the finding that the course was suitable for achieving the learning objectives and showed that it was also suitable for the participants to improve their self-, project, and team management skills. Overall, the results indicate that the course concept was well designed, implemented, and perceived and accepted positively.

### Perceived Importance of the Course to the Participants

The results showed that many students perceived the achievement of the superordinate learning objectives as important to them and that only 10% (1/10) of the participants felt that the course was irrelevant. This perception aligns with the aforementioned finding that many medical students want the topic of digital medicine to be (more extensively) integrated into their studies [11,16], supporting the relevance of offering this course. However, as the perceived importance of the course was not further recorded or classified, the findings should be interpreted with caution.

### Course Concept

#### Alignment of the Course With Findings From Teaching and Learning Research

From a pedagogical research perspective, the Bielefeld model integrates elements from multiple learning theories, including project-based learning, which has demonstrated efficacy [26], and authentic learning [27]. These approaches offer significant benefits as they may enhance the ability to recall knowledge in real-world problem-solving contexts [28] while also positively impacting student motivation and engagement [29]. In addition, in the Bielefeld model, theoretical and practical components were interwoven. Tempelman and Pilot [30] argue against the background of the theory of constructivism that this might help build knowledge and skills upon one another. In addition, structuring a course in which each unit builds on the previous one, as seen in the Bielefeld model, can promote a more cohesive learning experience. This can be beneficial for learning [31]. Medical education research has shown that the mix of knowledge and skill teaching in simulation-based medical education can be successful, with high effect sizes for learning outcomes of both aspects [32,33]. In light of these findings, the direct link between the theoretical and practical units in the Bielefeld model may enable participants to practically apply newly acquired knowledge directly and, thus, achieve the goal of holistically developing digital applications. An advantage of developing content in practical sessions, as implemented in the Bielefeld model, is that it makes learning outcomes more tangible, as Kuhn et al [34] point out. This approach may help in demonstrating learning success in a more verifiable and substantial way. The positive effect of including guest lecturers in the course has also been scientifically proven (eg, [35]). The idea of having medical students work on solutions for practical use cases at the interface between medicine and technology and in collaboration with students from other disciplines has already been implemented in a course described by Breil et al [36]. However, this was not geared toward digital medicine. In that course, medical students collaborated with computer science students. For this to work, the students had to develop soft skills such as communication skills and subject-specific knowledge. Almost all the participants in the aforementioned course rated the integration of theory and practice as good or excellent. Although it is unclear what exactly supported the good integration of theory and practice in that course, it can be assumed that this is generally related to the course concept. Therefore, the results suggest that the Bielefeld model, which followed a similar concept, facilitated a good integration of theory and practice as well. However, some aspects could be improved to align with findings from teaching and learning research. For example, although it was pointed out to which life cycle phases the topics of the individual sessions could be assigned in the Bielefeld model, this could have been explained more clearly. This might have helped make the course even more coherent (eg, [31]).

#### Alignment of the Course With Recommendations for Digital Medicine Courses

The design of the course is supported by recommendations and perspectives in the literature for implementing courses in digital medicine. The interdisciplinary design of the course aligns with the suggestion by Foadi and Varghese [37] that courses in digital medicine should be interdisciplinary due to the significant role that interdisciplinarity plays in the field of digital medicine. The Bielefeld model covered various topics, such as ethical aspects, legal frameworks, and entrepreneurial opportunities concerning digital medicine applications. Bahagon and Jacobson [2] state that successful implementation of eHealth solutions requires expertise in such areas. Students would like for courses on digital health to include precisely these aspects as well as practical training, for example, on developing apps, as Machleid et al [16] found out. Even though it cannot be said with certainty that these were also the wishes of the students enrolled in our course, it can be assumed based on this research finding. Therefore, in that case, these desires were well met by the course concept. Such a match between the students’ wishes and the course concept could explain why many of the students perceived the course as important to them and enjoyed and benefitted from it. According to Goldsack and Zanetti [38], clinical and technical expertise must be considered together for a successful digital transformation. In the Bielefeld model, medical students do not receive in-depth technical training. Nonetheless, they learn how to collaborate with people from the technical field and gain some insights into the technical implementation of digital medicine concepts. The University Digitalization Forum (Hochschulforum Digitalisierung [9]) points out that it is insufficient to teach only technical skills to improve digital transformation and that qualifications in areas such as communication and leadership or constitutional corporate design should also be provided. Although the Bielefeld model does not focus on this, it can be assumed that communication and leadership skills are also being learned during the teamwork units of the course.

As the course concept was geared precisely toward designing a telemedicine application and taking the framework conditions—various interdisciplinary factors—into consideration, it can be assumed that the learning objective of the NKLM stating that students should acquire the competencies to explain the application scenarios for telemedicine applications and their framework conditions [12] was achieved. In addition to this, other authors have described different frameworks regarding what students should learn about digital medicine. Brunner et al [39], for example, describe a framework that stipulates that students in the health care sector should achieve learning objectives related to the areas of (1) digital technologies, systems, and policies; (2) clinical practice and applications; (3) data analysis and knowledge creation; and (4) system and technology implementation. The Bielefeld model covers these areas at least to some extent, albeit more on a theoretical than a practical level. Kuhn [40] points out that, against the backdrop of digital transformation, physicians must be able to understand and categorize the change processes and new digital treatment concepts associated with change, and in addition to learning practical skills, they must also reflect on an attitude toward digital medicine. Due to the wide range of topics covered by the Bielefeld model, this is assumed to have been achieved at least to some extent. Recently, experts identified 40 specific digital health topics from the areas of knowledge, skills, and attitudes that they believe should be taught during medical school [41]. The Bielefeld model covers some of these knowledge and attitude topics.

In total, the Bielefeld model has many similarities with recommendations for the design of teaching in digital medicine. However, there are some aspects that could be given greater consideration in the Bielefeld model in the future to be even more in line with the recommendations. Including other experts or professional groups could be useful. In line with the suggestion that clinical and technical expertise is important for digital transformation [38], including cybersecurity experts or hardware and software engineers, for example, has been proposed [38]. It could also be useful to involve other groups, such as data scientists, ethicists, and patients [38]. To acquire and develop further knowledge and skills and a deeper attitude regarding digital medicine, it might make sense to include more content in the course and allow more time for a critical examination of digital medicine. However, as this is covered by other compulsory courses within the Bielefeld medical degree program, there is no need for the Bielefeld model to cover these aspects as well.

Furthermore, the question arises on whether the didactic formats used in the course correspond to what students want in digital medicine courses. Vossen et al [19] found that many students would like to be taught about technological developments through real-life scenarios and case descriptions. The Bielefeld model aims in this direction. Teaching in the form of lectures, which was also integrated into the Bielefeld model, was, on average, neither strongly supported nor strongly rejected in the study by Vossen et al [19]. Although, based on these results, this is not the preferred teaching format, it was necessary for the Bielefeld model to impart basic knowledge by offering lectures. The teaching format that received the most support in the study by Vossen et al [19] was remotely following a real-life patient under the supervision of a physician. However, such a teaching format is inappropriate for what needs to be taught and learned in the Bielefeld model. Therefore, the teaching format in the Bielefeld model cannot be meaningfully compared with it.

On the basis of the positive and improvable aspects mentioned in this section, the Bielefeld model will be continued, expanded, and further developed. Following the suggestion that the rapid pace of transformation processes must be taken into account and that it must be possible to adapt the specific curriculum, the Bielefeld model will be continuously refined and updated to adequately reflect current developments in digital medicine and general teaching and learning research.

### Student Education in Digital Medicine in Germany

#### Comparison of the Course With Courses in Digital Medicine or Digital Health in Germany

In this section, the similarities and differences between the Bielefeld model and courses in digital medicine or digital health at other locations in Germany will be analyzed. The Bielefeld model was only compared with courses with a similar focus in terms of content and for which a publication could be found in a literature search. It should be noted that works such as those by Aulenkamp et al [17] or Behrends et al [42] and internet research indicate that there are also other courses on digital health and medicine. As no publications were available for these courses or they were unsuitable for the comparison due to different framework conditions, they were omitted. Although some course concepts described by Aulenkamp et al [17] appear to be related to the courses included in the comparison, it cannot be said with certainty that this is the case. Courses that focus on artificial intelligence or machine learning or place a clear emphasis on topics from the field of medical informatics (eg, hospital information systems) are regarded as connected to digital medicine but were not included in the comparison because they have a completely different focus in terms of content from that of the Bielefeld model. The Bielefeld model was compared to the courses described by the following authors: (1) Ehlers et al [43]; (2) Werner et al [44]; (3) Chaltikyan et al [45]; (4) Behrends et al [42]; (5) Offergeld et al [46]; (6) Nitsche et al [47,48], whereby both works share the same course concept but differ in the names and number of modules and, therefore, probably in the specific content; (7) Poncette et al [49] and Seemann et al [50]; and (8) Kuhn et al [34], Kuhn [40], Kuhn and Jungmann [51], and Kuhn et al [52-54], whereby they all share the same course concept but differ slightly in the names and number of modules and, therefore, possibly in the specific content.

If several publications were found for a course, only one is discussed here if the information used for the comparison was sufficiently clear.

The comparison of the Bielefeld model with other courses did not reveal any immediately comparable courses. However, there were certain overlaps in the design. As in the Bielefeld model, a common feature of many course concepts is the use of guest lecturers from different areas [34,43,44,47,49] (eg, app developers and representatives of the state data protection authority [34], an industry panel with representatives from the biomedical field [44], and lecturers from hospital departments and academic and nonacademic institutions who lectured on interdisciplinary topics [44]). Similarly to the Bielefeld model, topics covering economic, legal, or ethical aspects were incorporated into some courses, such as those described by Poncette et al [49] or Ehlers et al [43]. Another feature that the Bielefeld model has in common with some other courses is combining theoretical and practical units [34,44,49]. A difference between the Bielefeld model and many courses with which it was compared is that many courses followed a structure in which different application areas and examples of digital medicine were often considered in various sessions [34,43,44,47,49]. In contrast, the Bielefeld model is a course in which participants design a digital application independently and continuously throughout the course. The objective was to enable students to develop holistic digital applications independently and become active as future designers of digital medicine. Concepts with which the Bielefeld course can be most closely compared in terms of this focus are the modules described by Poncette et al [49] or Seemann et al [50] and an orientation module described by Werner et al [44]. Although these modules also follow a basic structure that includes numerous different areas of application and examples, one similarity to our course concept is that the focus is on students designing a product for a specific problem in the health care system [49] or developing a business model for digital transformation [44]. This allows them to take on the role of various stakeholders in the health care system [49] or company representatives [44]. A difference between the Bielefeld model and the course described by Poncette et al [49] is that, in the latter, the practical units were individual sessions instead of being integrated into each session as in the Bielefeld model. They seem to be rather separate from the theoretical units. There is no information on this with regard to the module presented in the work by Werner et al [44]. In light of the previously discussed possible advantages that could arise from interweaving theoretical and practical units and building units on each other [30,31], in our opinion, directly linking theoretical and practical units might be more advantageous. Examining numerous application areas and examples of digital medicine, as provided for in the aforementioned courses, is certainly enriching for students to gain a broad overview of digital medicine. However, at Bielefeld University, the students already deal with these aspects as part of an earlier course in digital medicine during their studies.

In summary, it can be said that the Bielefeld model is a newly developed part of a series of courses that contributes to fulfilling the call to train medical students in digital medicine. The Bielefeld model follows a detailed concept that has not yet been found in other evaluated courses in Germany.

#### Challenges and Initiatives Regarding the Implementation of Digital Medicine Courses in Germany

Various challenges that can make the integration of digital health and medicine into medical education more difficult [55] must be addressed to offer even more courses in digital medicine nationwide. For example, a sufficient teaching staff with the relevant expertise is needed. However, unlike Bielefeld University, few universities currently have a professorship or chair for this area [55]. There are various approaches to solving these challenges [56]. Initiatives such as the Digitalization of Departments-Medicine working group (Digitalisierung in den Fächern – Medizin), which is part of the University Digitalization Forum, are, for example, developing or presenting solutions [9]. Several initiatives also drive the implementation of educational projects. For example, a reform project in digital medicine [40] was funded by the Stifterverband initiative as part of its joint Curriculum 4.0 program with the Carl Zeiss Foundation. In the field of medical informatics associated with digital medicine, educational programs are also being offered as part of the HiGHmed initiative, which began as part of the Medical Informatics Initiative [57]. Such offers can also enable the teaching of digital medicine. The creation and implementation of initiatives is seen as an important measure [56].

### Limitations

The interpretability of the results of evaluating the Bielefeld model is limited due to several aspects. The evaluation questionnaire was not subjected to a pilot test. Although it was designed based on largely careful considerations and reflected upon between the authors, a pilot study would have been favorable to determine its quality and suitability for evaluating the course concept. Although an attempt was made to ensure the validity of the items by carefully formulating them based on detailed considerations, the criterion and construct validity in particular were not statistically tested. Therefore, no reliable statement can be made regarding the existence of criterion and construct validity and, thus, external validity overall. The existence of retest reliability could have been examined to better determine the stability of the instruments, a facet of reliability. As this did not happen, it cannot be said with certainty whether the instruments are reliable in this regard. One limitation with regard to the design of the survey is that the achievement of the superordinate learning objectives was only recorded in the postsurvey. Therefore, no direct statement can be made as to whether they were achieved better after the course than before. Because the data were not analyzed using inferential statistics, the results cannot be reliably transferred to the general population. The interpretability and generalizability of the results are also limited by the small sample size and by potential biases that may have occurred. With regard to the sample, it should be taken into account that the Medical School OWL was only just commissioned in 2021 and that the Digital Medicine course presented in this paper was the first of its kind ever offered at this medical school. Therefore, it was not possible to form a larger sample by summarizing and analyzing data from several courses. In addition, the teachers had no influence on the number of course participants. Therefore, an evaluation based on the existing sample of 10 participants was the only option.

It was found that only two-thirds of the students who took part in the presurvey (10/15, 67%) also took part in the postsurvey. It is possible that the third who dropped out had little interest in digital medicine and, therefore, were not motivated enough to participate in the postsurvey. The sample would then only consist of students who were interested in the topic. In this case, the results would only be transferable to students with an interest in digital medicine instead of all medical students. It is also possible that the students with a particular interest in the topic also achieved the learning objectives better and rated the course better or that, in general, only students who felt that they had achieved the learning objectives participated in the postsurvey. Both would result in a systematic bias of the results. This is aggravated by the fact that the data were collected via the self-report of the students. If an objective measure of data collection had been used that did not involve any extra effort for the students, it might have been easier to motivate all students to provide data for the postevaluation. Such an objective measure could have been, for example, an analysis of the project outlines.

In addition to the aforementioned aspects, it was observed during numerous sessions of the course that some students were absent, and some sessions were only held on the web and the instructors do not know whether the participants worked on the topics of those sessions by themselves. It is questionable whether the students who were not physically present also improved in the learning objectives of these sessions. If this were the case, the question of what, if not the course, could have led to this improvement would arise. The data showed that not every learning objective was achieved better by all students after the course than before. This is particularly understandable if the students who were not present in the session covering this learning objective did not deal with the topic. However, these questions were not investigated.

The results of this evaluation should only be seen as initial indications of the effectiveness of the course concept in achieving the learning objectives due to the named limitations.

In addition, as part of the study, participants were asked to what extent they perceived various potential strengths of the course as such. This format made it possible to ask about specific aspects but has the disadvantage that other possible strengths of the course could remain hidden. Although we included an optional free-text field at the end of the surveys meant to allow participants to name strengths and additional aspects related to the course, it cannot be assumed that all participants used this option. A final statement on whether a course similar to the Bielefeld model already exists at other locations in Germany cannot be made as the course was only compared with course concepts for which a publication was found.

### Implications

Our results indicate that using the Bielefeld model might improve self-sufficiency in digital medicine instead of letting future physicians passively consume digital medicine’s offers. However, due to the small sample size and other limitations, this warrants additional evaluations. The observation made during the course that the medical and electrical engineering students learned what is essential when working with students from another discipline suggests that the course can be offered across disciplines and universities.

### Outlook

As inferential statistics were not performed, no direct conclusions can be drawn from this study on whether the same results can also be expected in the general population. In future studies, the concept should be implemented with and analyzed based on more participants and then subjected to an evaluation including inferential statistical procedures. Bielefeld University plans to continue conducting and evaluating the concept over the next few years to obtain a longitudinal sample with a larger number of cases and carry out inferential statistical analyses. To better assess whether the course contributes to the achievement of the superordinate learning objectives, their achievement should be recorded in future studies in the pre- and postsurvey. A more objective assessment of the achievement of the learning objectives could be made by analyzing the students’ project outlines. The project outlines make it clear whether the students know the factors that are relevant in the development of a digital application and whether they were able to apply them to their specific project.

### Conclusions

A novel approach to teaching digital medicine was conceived, executed, and assessed for the first time. The evaluation outcomes suggest that the course framework has the potential to effectively facilitate the transformation of participating students into future architects of digital medicine. These results signify that the course is poised to play a pivotal role in fostering digital transformation and seamlessly incorporating digital medicine into the medical curriculum, aligning with the aspirations of diverse stakeholders.

## Appendix

Multimedia Appendix 1 Overview of the three instructional cases for medical students, created by the course instructor.

Multimedia Appendix 2 Overview of web-based and in-person course sessions, including guest lecturers and main content, with additional materials and exercises provided.

Multimedia Appendix 3 Template of questions and content that students can consider when writing the project outline.

Multimedia Appendix 4 Schedule of a standard course session. The exact sequence of the individual sessions could be flexibly adapted.

Multimedia Appendix 5 Evaluation items overview. Items sub01 to sub17 and COM01 were included in both pre- and postsurveys, while others were postsurvey only.

Multimedia Appendix 6 Postsurvey response frequencies for evaluating learning objectives, course suitability, importance, enjoyment, benefit, and strengths.

Multimedia Appendix 7 Response frequencies for items evaluating objective achievement of subordinate learning objectives in the pre- and postsurvey.

Multimedia Appendix 8 Raw data for each participant on objective achievement of subordinate learning objectives in the pre- and postsurvey, including intraindividual changes.

## Abbreviations

NKLM: National Competency-Based Catalog of Learning Objectives
OWL: Ostwestfalen-Lippe
TUHH: Hamburg University of Technology
