# Supplementary material for: Teaching Digital Medicine to Undergraduate Medical Students With an Interprofessional and Interdisciplinary Approach: Development and Usability Study
Source: JMIR Med Educ. 2024 Sep 30;10:e56787. doi: 10.2196/56787 (PMC11474112; doi:10.2196/56787)
Supplement: Multimedia Appendix 1 [file mededu_v10i1e56787_app1.docx]

Table S1. Description of the three cases the students worked on during the course. The cases are formulated as an instruction for medical students. The cases were created by the main instructor of the course.

| **Case number** | **Case** |
| --- | --- |
| 1 | “Design a concept for the home-based, telemedical monitoring of patients with heart failure and reduced systolic pumping capacity (HFrEF). Consider the needs of the patients and physicians involved in the monitoring, aspects of the data infrastructure and carefully weigh up the selection of biomarkers to be measured.” |
| 2 | ”Design a concept for the home-based, telemedical monitoring of patients with advanced chronic obstructive pulmonary disease (COPD). Take into account the needs of patients and physicians involved in monitoring, aspects of the data infrastructure and carefully consider the selection of biomarkers to be measured." |
| 3 | “Design a system for monitoring critically ill patients that supports the model of the tele-emergency doctor in the rescue service. Here, paramedics or emergency paramedics are on site in the event of an emergency situation, while a tele-emergency doctor monitors the situation from a control center and issues medical instructions.” |
