# Supplementary material for: Teaching Digital Medicine to Undergraduate Medical Students With an Interprofessional and Interdisciplinary Approach: Development and Usability Study
Source: JMIR Med Educ. 2024 Sep 30;10:e56787. doi: 10.2196/56787 (PMC11474112; doi:10.2196/56787)
Supplement: Multimedia Appendix 2 [file mededu_v10i1e56787_app2.docx]

Table S2. Description of the individual course sessions that took place online or in person and the guest lecturers, if a guest lector was present in the respective session. The main contents of the individual sessions are presented. In addition to these, the students were usually provided with further information, use cases or optional exercises during the lectures or workshops.

| **Session** | **Main content of the respective session** | **Guest lecturer** |
| --- | --- | --- |
| Session 01 – Introduction | The students were told about the basics of digital medicine, for example what digital medicine is, what possible applications there are, or what challenges can arise in the development and implementation of digital medicine applications and how these can be addressed. Initial factors influencing the sustainable implementation of digital medical products and processes were outlined that would be covered in more detail during the course (e.g. data protection, interoperability, quality). A specific example of an application of digital medicine, an app-based rhythm monitoring after atrial fibrillation ablation, was presented.  The students were also introduced to the course concept, i.e. the course schedule was explained to them, they were divided into groups and assigned one of the three cases. They had the time to familiarize themselves with their case and the sensor they should use when solving it. | -^a^ |
| Session 02 - Project management | Students were explained how a project can be planned and managed. The guest lecturer explained various elements that need to be taken into consideration in order for the project to be implemented successfully. Aspects include, for example, determining stakeholders and identifying potential risks that could jeopardize the project. The students could try to plan and manage their project in a structured way with a project transfer report. | A professional project manager |
| Session 03 - Market entry | The students were told about the possibilities of bringing digital medicine applications to market in Germany, what needs to be taken into account when planning the market entry and which challenges the market entry and distribution of digital medicine applications can entail. As an example, they learned about the area of digital health applications that can be prescribed by prescription in Germany. | A consultant who advises international clients regarding market entry of digital medicine products |
| Session 04 -Technical development | The students were told what measurement means and that there are standard units of measurement. It was also explained to them that there can be uncertainties when measuring, which can arise from various sources of error and which can limit the interpretability of measurement results.  For the following collaboration with the electrical engineering students, the students should draft a technical requirement profile for the sensor used in their project. This should include information such as which measured values the sensor should record, how often it should measure, etc. This requirements analysis was then passed on to the electrical engineering students, who had to try to design the sensor according to the requirements. | A professor for smart sensor development. This is the professor of the electrical engineering students the medical students worked with. |
| Session 05 – Quality | The students were taught how to properly assess the quality of an application of digital medicine and how it is important for doctors to be able to assess the quality, not only in order to be able to treat patients well, but also in the context of liability issues. | -^a^ |
| Session 06 - Data science | Content relating to data science was available to students only for self-study on the course's online platform. The students were taught what data and data sources exist and how to evaluate them, to what extent large amounts of data can be used for personalized medicine and what distinguishes them. In addition, they were explained what challenges can be in data management and were taught initial knowledge on the subject of data analysis. They also got a brief insight into the subject of AI and machine learning.  The students were shown how simple data analyses can be carried out using Excel and how to visualize data. They were able to try out data processing with Excel themselves. | A data scientist working in a clinical cancer registry |
| Additional session A – Meeting with the electrical engineering students | The medical and the electrical engineering students discussed the technical requirements that the sensor used in the projects should fulfill and how well this can and could be implemented technically. | -^a^ |
| Session 07 - Interoperability | The students were explained what interoperability is, who in Germany takes care of interoperability in the healthcare sector and how efforts are made to implement interoperability. Using the example of a cancer registration, the topic was illustrated and possible challenges were addressed. | A person who is internationally involved in the topic of interoperability in cancer registration |
| Session 08 - Usability | The students were explained what is meant by usability, why it is important (mainly related to the field of digital medicine applications), how it can be tested and what to consider in the context of usability. | A professor for health informatics with focus on usability and user experience |
| Session 09 – Law, Regulation, Ethics | Students were explained how the development and implementation of digital medicine applications is legally regulated in Europe and Germany. They were explained that, from a legal perspective, certain medical products must be certified as medical devices. They have also received information on market access and liability.  Content relating to ethics was available to students only for self-study on the course's online platform. The students were introduced to the topic that the use of health-related mobile technology increases the level of autonomous use and that ethical considerations are relevant for utilisation decisions. Students were taught about the multidimensional ethical aspects that arise when using smartphones and health apps and their inherent needs, e.g. the need for privacy and transparency and the desire for freedom and control at the same time. In addition, the students were shown the tension between the collection, analysis and use of large amounts of medical data on the one hand and privacy, self-determination and other facets at the level of personal interests of patients and ethics on the other. | A lawyer in the field of medical law |
| Session 10 - Sex and gender sensitivity | The students were taught why sex and gender play a role in the development of digital innovations. For example, it was presented what sex and gender differences may be present in different diseases like the chronic obstructive pulmonary disease (COPD) and how the successful use of digital medicine applications may depend on sex and gender. | A professor for sex- and gender sensitive medicine |
| Session 11 - Data protection and data security | Content relating to data protection and security was available to students only for self-study on the course's online platform. The students were explained what the topics of data protection and data security are all about and learned about the legal basis. They were presented with why data protection and data security are relevant in the field of digital medicine, how they can be produced and what challenges there may be. They were also introduced to the question of what advantages and disadvantages the collection and analysis of personal data can have for individuals and what social developments can arise from this. | -^a^ |
| Session 12 - End | The students were able to reflect on the course and received information about the examination performance. | -^a^ |
| ^a^Not applicable. | | |
