# Supplementary material for: Teaching Digital Medicine to Undergraduate Medical Students With an Interprofessional and Interdisciplinary Approach: Development and Usability Study
Source: JMIR Med Educ. 2024 Sep 30;10:e56787. doi: 10.2196/56787 (PMC11474112; doi:10.2196/56787)
Supplement: Multimedia Appendix 3 [file mededu_v10i1e56787_app3.docx]

Table S3: Template of questions and content that students can consider when writing the project outline.

The template is formulated as an instruction for the students. The students could use this as a guide when writing their project outline. Students could enter their case number for the keyword “group”.

| Sample project outline: | | |
| --- | --- | --- |
| Version: [No.] [Date] | | |
| Title: |  | |
| Group: |  | |
| Group members: |  | |
| 1 | Idea | Notes |
| 1.1 | Name your target group (who is your product aimed at). |  |
| 1.2 | Explain the need. Describe the specific needs of the target groups that are met by the product. Name the main problem that will be solved |  |
| 1.3 | Name the goal of your project. |  |
| 1.4 | Describe what your project is about. Describe the value proposition, features and possible applications of your product idea. |  |
| 1.5 | Explain the relevance of the project. Explain what added value/enrichment the project has, e.g. for your target group. Elements can be, for example: What medical need is being addressed? Who will primarily benefit from the results achieved? What impact will the expected results have on the treatment of the disease addressed? To what extent will added value be created in comparison to existing solutions or solutions currently under development? |  |
| 2 | Describe the idea from a market perspective (also use internet research if this helps you). |  |
| 2.1 | Describe the international status of research and development with regard to solutions to the problem. |  |
| 2.2 | Identify and describe comparable competing products on the market. |  |
| 2.3 | Describe the unique selling proposition of your product (name features that make your product unique). Explain to what extent your project goes beyond the current international state of research and development / to what extent your product is innovative. |  |
| 2.4 | List the advantages and disadvantages of your product. |  |
| 2.5 | Estimate the market potential/market opportunities for your product. |  |
| 3 | Describe the idea from an implementation perspective. |  |
| 3.1 | Describe the technical solution approach (function, components, interfaces, etc.) and, if necessary, any necessary activities. |  |
| 3.2 | Describe whether and to what extent your product has a high usability and what might need to be changed in order to increase usability. |  |
| 3.3 | Describe whether a sex- and gender-sensitive design of your product is necessary, whether and in which way your product has a sex- and gender-sensitive design and what might need to be changed in order to increase sex and gender sensitivity. |  |
| 3.4 | Describe the extent to which interoperability exists and what might need to be changed in order to increase interoperability. |  |
| 3.5 | Describe the challenges and risks as well as points (technical, economic, (data protection) legal, ethical, regulatory, etc.) that must be paid particular attention to in order to ensure success. Explain how you meet the challenges and what you might need to change in order to solve them optimally (example: how well are ethical concerns taken into account and appropriate solutions found?) |  |
| 3.6 | Name existing internal or external property rights. (A basic search at the German Patent and Trade Mark Office can provide initial information). |  |
| 4 | Work objectives and methodology |  |
| 4.1 | Describe important to-do's/milestones from your point of view. Describe your work objectives and their methodical implementation. |  |
| 4.2 | Describe the partners you are working with to implement your project. |  |
