# Supplementary material for: Teaching Digital Medicine to Undergraduate Medical Students With an Interprofessional and Interdisciplinary Approach: Development and Usability Study
Source: JMIR Med Educ. 2024 Sep 30;10:e56787. doi: 10.2196/56787 (PMC11474112; doi:10.2196/56787)
Supplement: Multimedia Appendix 4 [file mededu_v10i1e56787_app4.docx]

Table S4. Schedule of a standard course session. The exact sequence of the individual sessions could be flexibly adapted. P=preparation, Lt=lecture time, Wt=workshop time, St=self-study time, Opt=optional offer, ∆ [Minutes]=duration of each activity in minutes.

| Status | Clock time (pm) | ∆ [Minutes] | Activity |
| --- | --- | --- | --- |
| P | 2:45 | 15 | Students arrive |
| Lt | 3:00 | 15 | Welcome, questions about the previous course day, introduction of guest (if applicable) |
| Lt | 3:15 | 30 | (Guest) Lecture |
| Lt | 3:45 | 5 | Comprehension questions about the lecture |
| Lt | 3:50 | 10 | Use Case presentation^b^ |
| Wt | 4:00 | 15 | Discussion presentation and use case |
| Wt | 4:15 | 15 | Pause |
| Wt | 4:30 | 45 | Group work: "project outline", consultation |
| Wt | 5:15 | 10 | Concluding discussion, conclusion |
| Wt | 5:25 | 5 | Outlook for next course day |
| Wt | 5:30 | -^a^ | Dismissal of students |
| St | 5:30 | 45 | Guided self-learning |
| Opt | 5:30 | 30 | Optional: Continuation of group work and respectively or debriefing. |
| St | 6:15 | -^a^ | End of the course day |
| ^a^Not applicable.  ^b^The instructor or guest lecturer, respectively, could present a use case on the topic of the lesson. Alternatively, the time could also be used for the presentation, questions or a discussion. Alternatively, the next activities could be brought forward. | | | |
