# Supplementary material for: Teaching Digital Medicine to Undergraduate Medical Students With an Interprofessional and Interdisciplinary Approach: Development and Usability Study
Source: JMIR Med Educ. 2024 Sep 30;10:e56787. doi: 10.2196/56787 (PMC11474112; doi:10.2196/56787)
Supplement: Multimedia Appendix 5 [file mededu_v10i1e56787_app5.docx]

Table S5. Overview of the items used in the evaluation. The items sub01 to sub17 and item COM01 were used in the pre- and post-survey. The other items were only used in the post-survey. ID=item identifier, sub=subordinate (learning objective), SUPER=superordinate (learning objective), OA=objective achievement (of the respective superordinate learning objective), SUIT=suitability (of the course concept for achieving the respective superordinate learning objective), IMP=importance (of achieving the respective superordinate learning objective), SA=subjective achievement (of the respective superordinate learning objective), FUN=(the module was) fun, BEN=(obtaining a) benefit, STR=strengths (of the course), AB=absence (during course sessions), COM=comment.

| ID | Item stem and item | |
| --- | --- | --- |
|  | I can… | |
| sub01 |  | … identify players and stakeholders in the field of digital medicine. |
| sub02 |  | … name and reflect on overarching themes of digital medicine. |
| sub03 |  | … identify, address, and discuss problem areas (medical, technical, legal, ethical, and social) of digital medicine. |
| sub04 |  | … critically assess "digital medicine" and evaluate it based on its opportunities and challenges. |
| sub05 |  | … explain the product life cycle of "digital medicine" and plan an applied project based on it. |
| sub06 |  | … plan a concrete project with basic knowledge of project management. |
| sub07 |  | … identify markets for "digital medicine" and discuss challenges of market entry of digital applications. |
| sub08 |  | … identify and communicate the necessary information required for commissioning a technical development of digital applications. |
| sub09 |  | … explain the legal challenges of using digital tools and digital communication media in relation to "digital medicine". |
| sub10 |  | … explain the regulatory difference between a medical device and other products and explain legal consequences. |
| sub11 |  | … identify and discuss the ethical implications of digital medicine for patients, physicians, contributors, society, and the environment. |
| sub12 |  | … explain interoperability and know what characterizes interoperability. |
| sub13 |  | … assess the quality of digital medicine applications. |
| sub14 |  | … recognize the quality of the usability of a digital application and know which factors can influence the usability. |
| sub15 |  | … discuss elements of Data Science, its tools, and requirements in data preparation and data analysis. |
| sub16 |  | … discuss and evaluate "digital medicine" with regard to specific aspects in the context of gender and sex. |
| sub17 |  | … explain data protection and data security challenges in the development and use of digital tools and digital communication media. |
| SUPER1_OA |  | I know the factors that influence the sustainable implementation of digital medical products and processes. |
| SUPER1_SUIT |  | The course concept was suitable for teaching these factors. |
| SUPER1_IMP |  | It was important to me to achieve this learning goal. |
| SUPER1_SA |  | I have achieved this learning goal from a personal perspective. |
| SUPER2_OA |  | I can apply my knowledge regarding the factors that influence the sustainable implementation of digital medical products and processes in developing a concrete project. |
| SUPER2_ SUIT |  | The course concept was suitable to apply my knowledge regarding these factors in developing a concrete project. |
| SUPER2_ IMP |  | It was important to me to achieve this learning goal. |
| SUPER2_SA |  | I have achieved this learning goal from a personal perspective. |
| SUPER3_OA |  | I feel empowered to design sustainable digital products and processes in future projects. |
| SUPER3_ SUIT |  | The course concept was suitable to enable me to design sustainable digital products and processes in future projects. |
| SUPER3_ IMP |  | It was important to me to achieve this learning goal. |
| SUPER3_SA |  | I have achieved this learning goal from a personal perspective. |
| FUN01 |  | The 'Digital Medicine' module was fun to do. |
| BEN01 |  | I benefited from the 'Digital Medicine' module. |
|  | Strengths of the digital medicine course were: | |
| STR01 |  | The teaching staff (friendliness, openness, appreciation, professionalism, interdisciplinarity) |
| STR02 |  | The design of the courses (content preparation, interaction, material, equipment) |
| STR03 |  | Timing of classes (punctuality, time frame for lectures and seminars). |
| STR04 |  | Offline content and preparation in the LernraumPlus |
| AB01 |  | If I was unable to attend course days, it was largely due to the following reasons (multiple choices possible):   - Too little time capacity due to other study-related requirements - Insufficient time capacity due to personal demands - Illness - The course was irrelevant to me - Other |
| COM01 |  | Here you can enter further comments /suggestions / proposals |
