# Supplementary material for: Teaching Digital Medicine to Undergraduate Medical Students With an Interprofessional and Interdisciplinary Approach: Development and Usability Study
Source: JMIR Med Educ. 2024 Sep 30;10:e56787. doi: 10.2196/56787 (PMC11474112; doi:10.2196/56787)
Supplement: Multimedia Appendix 6 [file mededu_v10i1e56787_app6.docx]

Table S6. Frequency of the individual response categories regarding the items assessing the objective and subjective achievement of the three superordinate learning objectives, the suitability of the course for achieving them, the importance of achieving them, enjoyment and benefit obtained from the course and strengths of the course, applied in the post-survey. N=10 participants, n=number of participants who gave the respective answer, ID=item identifier, IQR=interquartile range, SUPER=superordinate (learning objective), OA=objective achievement (of the respective superordinate learning objective), SUIT=suitability (of the course concept for achieving the respective superordinate learning objective), IMP=importance (of achieving the respective superordinate learning objective), SA=subjective achievement (of the respective superordinate learning objective), FUN=(the module was) fun, BEN=(obtaining a) benefit, STR=strengths (of the course).

| ID | Item stem and item | | Strongly  disagree (1), n (%) | Rather  disagree (2), n (%) | Rather neutral (3), n (%) | Rather agree (4), n (%) | Strongly agree (5), n (%) | Median (IQR)^a^ |
| --- | --- | --- | --- | --- | --- | --- | --- | --- |
| SUPER1_OA |  | I know the factors that influence the sustainable implementation of digital medical products and processes. | 0 (0) | 1 (10) | 1(10) | 4(40) | 4(40) | 4 (4-5) |
| SUPER1_SUIT |  | The course concept was suitable for teaching these factors. | 0 (0) | 2 (20) | 0 (0) | 2 (20) | 6 (60) | 5 (4-5) |
| SUPER1_IMP |  | It was important to me to achieve this learning goal. | 0 (0) | 0 (0) | 4 (40) | 4 (40) | 2 (20) | 4 (3-4) |
| SUPER1_SA |  | I have achieved this learning from a personal perspective. | 0 (0) | 1 (10) | 2 (20) | 5 (50) | 2 (20) | 4 (3-4) |
| SUPER2_OA^b^ |  | I can apply my knowledge regarding the factors that influence the sustainable implementation of digital medical products and processes in developing a concrete project. | 0 (0) | 1 (11) | 2 (22) | 3 (33) | 3 (33) | 4 (3-5) |
| SUPER2_SUIT |  | The course concept was suitable to apply my knowledge regarding these factors in developing a concrete project. | 0 (0) | 0 (0) | 1 (10) | 2 (20) | 7 (70) | 5 (4-5) |
| SUPER2_IMP |  | It was important to me to achieve this learning goal. | 0 (0) | 1 (10) | 3 (30) | 3 (30) | 3 (30) | 4 (3-5) |
| SUPER2_SA |  | I have achieved this learning from a personal perspective. | 0 (0) | 1 (10) | 2 (20) | 2 (20) | 5 (50) | 4.5 (3-5) |
| SUPER3_OA^b^ |  | I feel empowered to design  sustainable digital products and processes in future projects. | 0 (0) | 1 (11) | 0 (0) | 6 (67) | 2 (22) | 4 (4-4) |
| SUPER3_SUIT |  | The course concept was suitable to enable me to design sustainable digital products and processes in future projects. | 0 (0) | 0 (0) | 2 (20) | 1 (10) | 7 (70) | 5 (4-5) |
| SUPER3_IMP |  | It was important to me to achieve this learning goal. | 0 (0) | 0 (0) | 5 (50) | 2 (20) | 3 (30) | 3.5 (3-5) |
| SUPER3_SA |  | I have achieved this learning from a personal perspective. | 0 (0) | 1 (10) | 2 (20) | 4 (40) | 3 (30) | 4 (3-5) |
| FUN01 |  | The ‘Digital Medicine’ module was fun to do. | 0 (0) | 0 (0) | 0 (0) | 4 (40) | 6 (60) | 5 (4-5) |
| BEN01 |  | I had a benefit from the ‘Digital Medicine’ module. | 0 (0) | 0 (0) | 1 (10) | 4 (40) | 5 (50) | 4.5 (4-5) |
|  | Strengths of the digital medicine course were: | | | | | | | |
| STR01 |  | The teaching staff (friendliness, openness, appreciation, professionalism, interdisciplinarity) | 0 (0) | 0 (0) | 0 (0) | 0 (0) | 10 (100) | 5 (5-5) |
| STR02 |  | The design of the courses (content preparation, interaction, material,  equipment) | 0 (0) | 0 (0) | 0 (0) | 3 (30) | 7 (70) | 5 (4-5) |
| STR03 |  | Timing of classes (punctuality, time frame for lectures and seminars). | 0 (0) | 1 (10) | 2 (20) | 1 (10) | 6 (60) | 5 (3-5) |
| STR04 |  | Offline content and preparation in the LernraumPlus | 1 (10) | 0 (0) | 1 (10) | 4 (40) | 4 (40) | 4 (4-5) |
| ^a^This column is identical to the corresponding column from Tables 5, 9 or 10, respectively, in the article. It has been added here for the sake of completeness.  ^b^This item was answered by only nine participants. | | | | | | | | |
