# Supplementary material for: Teaching Digital Medicine to Undergraduate Medical Students With an Interprofessional and Interdisciplinary Approach: Development and Usability Study
Source: JMIR Med Educ. 2024 Sep 30;10:e56787. doi: 10.2196/56787 (PMC11474112; doi:10.2196/56787)
Supplement: Multimedia Appendix 7 [file mededu_v10i1e56787_app7.docx]

Table S7. Frequency of the individual response categories regarding the items assessing the objective achievement of the subordinate learning objectives, applied in the pre- and post-survey. N=10 participants, n=number of participants who gave the respective answer, ID=item identifier, IQR=interquartile range, ∆Median=change in the median from the pre- to the post-survey, sub=subordinate (learning objective).

| ID | Strongly  disagree (1) | | Rather  disagree (2) | | Rather neutral (3) | | Rather agree (4) | | Strongly agree (5) | | Pre-survey score,  median (IQR)^a^ | Post-survey score,  median (IQR)^a^ | ∆Median ^a^ |
| --- | --- | --- | --- | --- | --- | --- | --- | --- | --- | --- | --- | --- | --- |
|  | Pre | Post | Pre | Post | Pre | Post | Pre | Post | Pre | Post |  |  |  |
|  | n (%) | n (%) | n (%) | n (%) | n (%) | n (%) | n (%) | n (%) | n (%) | n (%) |  |  |  |
| sub01 | 4 (40) | 1 (10) | 3 (30) | 0 (0) | 2 (20) | 2 (20) | 1 (10) | 4 (40) | 0 (0) | 3 (30) | 2 (1-3) | 4 (3-5) | 2 |
| sub02 | 1 (10) | 0 (0) | 3 (30) | 0 (0) | 2 (20) | 0 (0) | 3 (30) | 5 (50) | 1 (10) | 5 (50) | 3 (2-4) | 4.5 (4-5) | 1.5 |
| sub03 | 1 (10) | 0 (0) | 1 (10) | 0 (0) | 2 (20) | 0 (0) | 3 (30) | 6 (60) | 3 (30) | 4 (40) | 4 (3-5) | 4 (4-5) | 0 |
| sub04 | 0 (0) | 0 (0) | 1 (10) | 0 (0) | 3 (30) | 0 (0) | 4 (40) | 6 (60) | 2 (20) | 4 (40) | 4 (3-4) | 4 (4-5) | 0 |
| sub05 | 3 (30) | 1 (10) | 5 (50) | 1 (10) | 2 (20) | 1 (10) | 0 (0) | 6 (60) | 0 (0) | 1 (10) | 2 (1-2) | 4 (3-4) | 2 |
| sub06 | 3 (30) | 0 (0) | 5 (50) | 0 (0) | 0 (0) | 4 (40) | 2 (20) | 3 (30) | 0 (0) | 3 (30) | 2 (1-2) | 4 (3-5) | 2 |
| sub07 | 3 (30) | 0 (0) | 1 (10) | 0 (0) | 5 (50) | 3 (30) | 1 (10) | 4 (40) | 0 (0) | 3 (30) | 3 (1-3) | 4 (3-5) | 1 |
| sub08 | 2 (20) | 0 (0) | 4 (40) | 0 (0) | 3 (30) | 2 (20) | 1 (10) | 8 (80) | 0 (0) | 0 (0) | 2 (2-3) | 4 (4-4) | 2 |
| sub09 | 1 (10) | 0 (0) | 3 (30) | 0 (0) | 4 (40) | 0 (0) | 2 (20) | 8 (80) | 0 (0) | 2 (20) | 3 (2-3) | 4 (4-4) | 1 |
| sub10 | 2 (20) | 0 (0) | 1 (10) | 1 (10) | 6 (60) | 1 (10) | 1 (10) | 5 (50) | 0 (0) | 3 (30) | 3 (2-3) | 4 (4-5) | 1 |
| sub11 | 2 (20) | 0 (0) | 0 (0) | 1 (10) | 4 (40) | 4 (40) | 4 (40) | 3 (30) | 0 (0) | 2 (20) | 3 (3-4) | 3.5 (3-4) | 0.5 |
| sub12 | 5 (50) | 2 (20) | 2 (20) | 0 (0) | 2 (20) | 3 (30) | 1 (10) | 3 (30) | 0 (0) | 2 (20) | 1.5 (1-3) | 3.5 (3-4) | 2 |
| sub13 | 3 (30) | 0 (0) | 0 (0) | 0 (0) | 5 (50) | 2 (20) | 2 (20) | 5 (50) | 0 (0) | 3 (30) | 3 (2-3) | 4 (4-5) | 1 |
| sub14 | 3 (30) | 0 (0) | 3 (30) | 0 (0) | 2 (20) | 1 (10) | 2 (20) | 6 (60) | 0 (0) | 3 (30) | 2 (1-3) | 4 (4-5) | 2 |
| sub15 | 4 (40) | 1 (10) | 3 (30) | 3 (30) | 3 (30) | 2 (20) | 0 (0) | 4 (40) | 0 (0) | 0 (0) | 2 (1-3) | 3 (2-4) | 1 |
| sub16 | 3 (30) | 0 (0) | 3 (30) | 1 (10) | 3 (30) | 2 (20) | 1 (10) | 6 (60) | 0 (0) | 1 (10) | 2 (1-3) | 4 (3-4) | 2 |
| sub17 | 2 (20) | 0 (0) | 3 (30) | 0 (0) | 3 (30) | 4 (40) | 2 (20) | 4 (40) | 0 (0) | 2 (20) | 2.5 (2-3) | 4 (3-4) | 1.5 |
| ^a^This column is identical to the corresponding column from Table 6 in the article. It has been added here for the sake of completeness. | | | | | | | | | | | | | |
