# Supplementary material for: Teaching Digital Medicine to Undergraduate Medical Students With an Interprofessional and Interdisciplinary Approach: Development and Usability Study
Source: JMIR Med Educ. 2024 Sep 30;10:e56787. doi: 10.2196/56787 (PMC11474112; doi:10.2196/56787)
Supplement: Multimedia Appendix 8 [file mededu_v10i1e56787_app8.docx]

Table S8. Raw data for each participant for each item regarding the objective achievement of the subordinate learning objectives in the pre- and post-survey and intraindividual changes in the raw data from pre to post. N=10 participants, ID=item identifier, sub=subordinate (learning objective), ∆=change in the raw data from the pre- to the post-survey, ∆Median=change in the median from the pre- to the post-survey.

|  |  |  | **Individual Subjects** | | | | | | | | | |
| --- | --- | --- | --- | --- | --- | --- | --- | --- | --- | --- | --- | --- |
| **ID** | | **Category** | **1** | **2** | **3** | **4** | **5** | **6** | **7** | **8** | **9** | **10** |
|  | sub01 | pre | 3 | 2 | 1 | 1 | 2 | 3 | 1 | 4 | 2 | 1 |
|  |  | post | 4 | 3 | 1 | 4 | 5 | 5 | 4 | 4 | 5 | 3 |
|  |  | ∆ | 1 | 1 | = | 3 | 3 | 2 | 3 | = | 3 | 2 |
|  | sub02 | pre | 4 | 4 | 1 | 4 | 5 | 3 | 2 | 3 | 2 | 2 |
|  |  | post | 4 | 5 | 4 | 5 | 5 | 5 | 5 | 4 | 4 | 4 |
|  |  | ∆ | = | 1 | 3 | 1 | = | 2 | 3 | 1 | 2 | 2 |
|  | sub03 | pre | 4 | 5 | 2 | 5 | 4 | 5 | 1 | 4 | 3 | 3 |
|  |  | post | 4 | 5 | 5 | 4 | 4 | 5 | 4 | 4 | 5 | 4 |
|  |  | ∆ | = | = | 3 | -1 | = | = | 3 | = | 2 | 1 |
|  | sub04 | pre | 3 | 5 | 2 | 4 | 4 | 5 | 3 | 4 | 3 | 4 |
|  |  | post | 5 | 5 | 4 | 4 | 4 | 5 | 4 | 4 | 5 | 4 |
|  |  | ∆ | 2 | = | 2 | = | = | = | 1 | = | 2 | = |
|  | sub05 | pre | 2 | 2 | 1 | 1 | 3 | 3 | 1 | 2 | 2 | 2 |
|  |  | post | 4 | 4 | 1 | 4 | 5 | 4 | 3 | 4 | 4 | 2 |
|  |  | ∆ | 2 | 2 | = | 3 | 2 | 1 | 2 | 2 | 2 | = |
|  | sub06 | pre | 2 | 2 | 1 | 1 | 2 | 4 | 1 | 2 | 4 | 2 |
|  |  | post | 4 | 4 | 3 | 5 | 5 | 5 | 3 | 4 | 3 | 3 |
|  |  | ∆ | 2 | 2 | 2 | 4 | 3 | 1 | 2 | 2 | -1 | 1 |
|  | sub07 | pre | 3 | 3 | 1 | 2 | 3 | 3 | 1 | 4 | 3 | 1 |
|  |  | post | 4 | 4 | 3 | 5 | 3 | 5 | 5 | 4 | 4 | 3 |
|  |  | ∆ | 1 | 1 | 2 | 3 | = | 2 | 4 | = | 1 | 2 |
|  | sub08 | pre | 2 | 3 | 1 | 1 | 2 | 3 | 2 | 3 | 4 | 2 |
|  |  | post | 3 | 4 | 3 | 4 | 4 | 4 | 4 | 4 | 4 | 4 |
|  |  | ∆ | 1 | 1 | 2 | 3 | 2 | 1 | 2 | 1 | = | 2 |
|  | sub09 | pre | 4 | 3 | 3 | 2 | 2 | 3 | 1 | 4 | 2 | 3 |
|  |  | post | 4 | 5 | 4 | 4 | 5 | 4 | 4 | 4 | 4 | 4 |
|  |  | ∆ | = | 2 | 1 | 2 | 3 | 1 | 3 | = | 2 | 1 |
|  | sub10 | pre | 3 | 2 | 1 | 3 | 3 | 4 | 1 | 3 | 3 | 3 |
|  |  | post | 5 | 5 | 2 | 4 | 5 | 4 | 4 | 4 | 4 | 3 |
|  |  | ∆ | 2 | 3 | 1 | 1 | 2 | = | 3 | 1 | 1 | = |
|  | sub11 | pre | 4 | 4 | 1 | 4 | 4 | 3 | 1 | 3 | 3 | 3 |
|  |  | post | 3 | 5 | 2 | 3 | 4 | 5 | 3 | 4 | 3 | 4 |
|  |  | ∆ | -1 | 1 | 1 | -1 | = | 2 | 2 | 1 | = | 1 |
|  | sub12 | pre | 3 | 2 | 1 | 1 | 4 | 3 | 1 | 1 | 1 | 2 |
|  |  | post | 4 | 5 | 1 | 4 | 4 | 5 | 3 | 3 | 1 | 3 |
|  |  | ∆ | 1 | 3 | = | 3 | = | 2 | 2 | 2 | = | 1 |
|  | sub13 | pre | 3 | 3 | 1 | 4 | 3 | 4 | 1 | 1 | 3 | 3 |
|  |  | post | 4 | 5 | 4 | 5 | 4 | 5 | 4 | 3 | 3 | 4 |
|  |  | ∆ | 1 | 2 | 3 | 1 | 1 | 1 | 3 | 2 | = | 1 |
|  | sub14 | pre | 3 | 4 | 1 | 2 | 2 | 3 | 1 | 4 | 2 | 1 |
|  |  | post | 5 | 5 | 4 | 4 | 5 | 4 | 4 | 4 | 3 | 4 |
|  |  | ∆ | 2 | 1 | 3 | 2 | 3 | 1 | 3 | = | 1 | 3 |
|  | sub15 | pre | 3 | 2 | 1 | 2 | 1 | 3 | 1 | 2 | 3 | 1 |
|  |  | post | 4 | 4 | 1 | 4 | 2 | 4 | 2 | 2 | 3 | 3 |
|  |  | ∆ | 1 | 2 | = | 2 | 1 | 1 | 1 | = | = | 2 |
|  | sub16 | pre | 2 | 2 | 1 | 1 | 1 | 3 | 4 | 3 | 2 | 3 |
|  |  | post | 2 | 4 | 3 | 4 | 4 | 5 | 4 | 3 | 4 | 4 |
|  |  | ∆ | = | 2 | 2 | 3 | 3 | 2 | = | = | 2 | 1 |
|  | sub17 | pre | 2 | 3 | 1 | 4 | 4 | 3 | 1 | 3 | 2 | 2 |
|  |  | post | 4 | 3 | 4 | 5 | 4 | 5 | 3 | 3 | 4 | 3 |
|  |  | ∆ | 2 | = | 3 | 1 | = | 2 | 2 | = | 2 | 1 |
| **Scores** | |  |  |  |  |  |  |  |  |  |  |  |
|  | Pre-survey score, median (IQR)^a^ |  | 3 (2-3) | 3 (2-4) | 1 (1-1) | 2 (1-4) | 3 (2-4) | 3 (3-4) | 1 (1-1) | 3 (2-4) | 3 (2-3) | 2 (2-3) |
|  | Post-survey score, median (IQR)^a^ |  | 4 (4-4) | 5 (4-5) | 3 (2-4) | 4 (4-5) | 4 (4-5) | 5 (4-5) | 4 (3-4) | 4 (3-4) | 4 (3-4) | 4 (3-4) |
|  | ∆Median^a^ |  | 1 | 2 | 2 | 2 | 1 | 2 | 3 | 1 | 1 | 2 |
| ^a^This row is identical to the corresponding column from Table 8 in the article. It has been added here for the sake of completeness. | | | | | | | | | | | | |
